# Functional Analysis of Polyprenyl Diphosphate Synthase Genes Involved in Plastoquinone and Ubiquinone Biosynthesis in *Salvia miltiorrhiza*

**DOI:** 10.3389/fpls.2019.00893

**Published:** 2019-07-09

**Authors:** Miaomiao Liu, Yimian Ma, Qing Du, Xuemin Hou, Meizhen Wang, Shanfa Lu

**Affiliations:** ^1^Institute of Medicinal Plant Development, Chinese Academy of Medical Sciences & Peking Union Medical College, Beijing, China; ^2^Key Laboratory for Tibet Plateau Phytochemistry of Qinghai Province, College of Pharmacy, Qinghai Nationalities University, Xining, China

**Keywords:** plastoquinone, polyprenyl diphosphate synthase, prenyltransferase, *Salvia miltiorrhiza*, transgenics, ubiquinone

## Abstract

Polyprenyl diphosphate synthase (PPS) plays important roles in the biosynthesis of functionally important plastoquinone (PQ) and ubiquinone (UQ). However, only few plant *PPS* genes have been functionally characterized. Through genome-wide analysis, two *PPS* genes, termed *SmPPS1* and *SmPPS2*, were identified from *Salvia miltiorrhiza*, an economically significant Traditional Chinese Medicine material and an emerging model medicinal plant. SmPPS1 and SmPPS2 belonged to different phylogenetic subgroups of plant trans-long-chain prenyltransferases and exhibited differential tissue expression and light-induced expression patterns. Computational prediction and transient expression assays showed that SmPPS1 was localized in the chloroplasts, whereas SmPPS2 was mainly localized in the mitochondria. SmPPS2, but not SmPPS1, could functionally complement the *coq1* mutation in yeast cells and catalyzed the production of UQ-9 and UQ-10. Consistently, both UQ-9 and UQ-10 were detected in *S. miltiorrhiza* plants. Overexpression of *SmPPS2* caused significant UQ accumulation in *S. miltiorrhiza* transgenics, whereas down-regulation resulted in decreased UQ content. Differently, *SmPPS1* overexpression significantly elevated PQ-9 content in *S. miltiorrhiza*. Transgenic lines showing a down-regulation of *SmPPS1* expression exhibited decreased PQ-9 level, abnormal chloroplast and trichome development, and varied leaf bleaching phenotypes. These results suggest that *SmPPS1* is involved in PQ-9 biosynthesis, whereas *SmPPS2* is involved in UQ-9 and UQ-10 biosynthesis.

## Introduction

Plastoquinone (PQ) and ubiquinone (UQ, also known as Coenzyme Q, CoQ) are two major lipid-soluble prenylquinones involved in electron transfer and energy transformation in the plastids and mitochondria. PQs mainly present in higher plants, algae and cyanobacteria and are involved in photosynthesis, chlororespiration and carotenoid biosynthesis (Tian et al., 2007). UQs exist in all aerobic organisms, such as plants and animals, and serve not only as coenzymes but also, in the reduced form, as antioxidants (Bentinger et al., 2007). Both PQs and UQs have the structural feature of a medium/long trans-polyprenyl side chain attached to the benzoquinone skeleton. Prenyl side chains are formed by sequential condensation of isopentenyl diphosphate (IPP) with allylic diphosphate in trans-configuration. The side chain of UQs contains variable numbers of isoprenoid units in different species. For instance, the side chain of UQ in *Escherichia coli*, *Saccharomyces cerevisiae*, and *Schizosaccharomyces pombe* contains eight (UQ-8), six (UQ-6), and ten isoprenoid unites (UQ-10), respectively (Asai et al., 1994; Suzuki et al., 1997; Kainou et al., 2001; Saiki et al., 2003; Kawamukai, 2009). In plants, the side chain of PQ usually contains nine isoprenoid units, whereas UQ side chain can be nine or ten isoprenoid units (Ducluzeau et al., 2012; Jones et al., 2013). For instance, *Arabidopsis thaliana* and most cereal crops contain UQ-9, whereas some vegetables, fruits and berries contain both UQ-9 and UQ-10 (Kamei et al., 1986; Mattila and Kumpulainen, 2001).

Although PQ and UQ play important roles in organisms, long-chain prenyl diphosphate synthase genes involved in the biosynthesis of PQ and UQ side chains have been functionally characterized only in *A. thaliana*, *Oryza sativa*, *S. lycopersicum*, and *Hevea brasiliensis* (Hirooka et al., 2003, 2005; Jun et al., 2004; Phatthiya et al., 2007; Ohara et al., 2010; Ducluzeau et al., 2012; Jones et al., 2013). In *Arabidopsis*, the main contributor that makes the solanesyl moiety of UQ-9 is At2g34630, which is a mitochondrion-located enzyme (Bouvier et al., 2000; van Schie et al., 2007; Danesi et al., 2011; Ducluzeau et al., 2012), whereas the genes encoded solanesyl diphosphate synthase targeted to chloroplasts and involved in PQ formation are At1g78510 and At1g17050 (Jun et al., 2004; Ducluzeau et al., 2012). Rice solanesyl diphosphate synthase genes involved in UQ-9 and PQ formation are *OsSPS1* and *OsSPS2*, respectively. *OsSPS1* was highly expressed in roots and localized in the mitochondria, whereas *OsSPS2* was abundant in both leaves and roots and localized in the plastids (Ohara et al., 2010). *S. lycopersicum* solanesyl diphosphate synthase gene (*SlSPS*) and decaprenyl diphosphate synthase gene (*SlDPS*) are responsible for the production of PQ and UQ-10, respectively. *SlSPS* was targeted to the plastids and involved in PQ biosynthesis, while *SlDPS* could extend the prenyl chain length of UQ-6 to ten isoprenoid units and produce UQ-10 (Jones et al., 2013). In *H. brasiliensis*, *HbSDS* was involved in the synthesis of the prenyl side chain of PQ (Phatthiya et al., 2007). The *Hevea* solanesyl polyprenyl diphosphate synthase (PPS) gene involved in UQ formation has not been reported. Up to date, long-chain trans-type prenyltransferases genes related to PQ or UQ side chain biosynthesis in other higher plants remains unknown.

*Salvia miltiorrhiza* Bunge (Danshen in Chinese) is known for its great economic and medicinal value (Ma et al., 2012; Zhang and Lu, 2017). In recent years, it is emerging as a model medicinal plant for scientific research (Song et al., 2013; Deng and Lu, 2017). The genome of *S. miltiorrhiza* has been decoded (Zhang et al., 2015; Xu et al., 2016). In previous studies, we identified from the current *S. miltiorrhiza* genome assembly forty terpenoid biosynthesis-related genes, of which nine encode prenyltransferases (Ma et al., 2012). Based on the homologies of deduced amino acid sequences with other known prenyltransferases, the nine prenyltransferase genes identified from *S. miltiorrhiza* were predicted to encode short-chain prenyltransferases, including three geranylgeranyl diphosphate synthases (SmGGPPS1, SmGGPPS2, and SmGGPPS3), a farnesyl diphosphate synthase (SmFPPS), four heterodimeric geranyl diphosphate synthases (SmGPPS.LSU, SmGPPS.SSUI, SmGPPS.SSUII.1, and SmGPPS.SSUII.2), and a homodimeric geranyl diphosphate synthase (SmGPPS). To date, no *S. miltiorrhiza* genes encoding long-chain PPS have been characterized. Through genome sequence analysis and subsequent molecular cloning, we identified two long-chain prenyltransferases genes, termed *SmPPS1* and *SmPPS2*, respectively. Comprehensive analysis showed that *SmPPS1* were involved in PQ-9 biosynthesis, whereas *SmPPS2* were involved in the biosynthesis of UQ-9 and UQ-10.

## Materials and Methods

### Sequence Retrieval and Gene Identification

The amino acid sequences of plant solanesyl diphosphate synthases, including At1g17050, At1g78510 and At2g34630 in *Arabidopsis*, OsSPS1 and OsSPS2 in *O. sativa* and HbSDS in *H. brasiliensis*, were used for homologous search against the current assembly of *S. miltiorrhiza* (line 99–3) genome using tBLASTn algorithm in local server with an *E*-value cutoff of 1e-5 (Altschul et al., 1990, 1997; Xu et al., 2016). Exon/intron structures and coding sequences were predicted on the Genscan web server (Burge and Karlin, 1998)^1^ and corrected manually.

### Plant Materials and RNA Extraction

Plant materials used for tissue-specific expression and RACE experiments were 2-year-old, field-grown *S. miltiorrhiza* (line 99–3) plants. Flowers, leaves, stems, root cortices and root steles were collected and stored in liquid nitrogen until use. The plants used for analysis of light-induced expression of SmPPS1 and SmPPS2 were cultivated as described below. *S. miltiorrhiza* stem segments with nods and shoot tips were surface-sterilized and cultivated on MS agar media (Murashige and Skoog) at 25°C. The regenerated shoots were sub-cultured every 6 weeks. Four-week-old plantlets were transferred to soil and grown in a greenhouse for additional 4 weeks (18–30°C and about 14 h of light per day). Twenty uniformly growing shoots were selected for the analysis of light-induced expression of *SmPPS1* and *SmPPS2*. Leaves were collected every 4 h from 16:00 pm on June 11th to 12:00 pm on June 12, 2013, when the time for sunrise was about 4:45 and for sunset was about 19:43. Plant materials were stored in liquid nitrogen immediately after collection.

Total RNA was extracted from plant tissues using the plant total RNA extraction kit (BioTeke, China). Genomic DNA contamination was eliminated by pretreatment of total RNA with RNase-free DNase (Promega, United States). RNA integrity was analyzed on 1% agarose gel. RNA quantity was determined using a NanoDrop 2000C spectrophotometer (Thermo Fisher Scientific, United States).

### Rapid Amplification of cDNA Ends and Cloning of Full Length *SmPPS1* and *SmPPS2*

Total RNA isolated from roots of *S. miltiorrhiza* was used for mRNA purification using the Oligotex mRNA isolation kit (QIAGEN, Germany). First-strand cDNA was synthesized from the purified mRNA by SuperScript III reverse transcriptase (Invitrogen, United States) as per the manual. 5′ and 3′ RACE experiments were performed using the GeneRacerTM Kit (Invitrogen, United States). Gene-specific nesting primers used for 5′ and 3′ RACE of *SmPPS1* are SmPPS1-R3 and SmPPS1-F1, respectively. Gene-specific nested primers used for 5′ and 3′ RACE of *SmPPS1* are SmPPS1-R2 and SmPPS1-F3, respectively (Supplementary Table S1). Gene-specific primers used for 5′ and 3′ RACE of *SmPPS2* are SmPPS2-R3 (5′ RACE nesting primer), SmPPS2-F2 (3′ RACE nesting primer), SmPPS2-R2 (5′ RACE nested primer), and SmPPS2-F3 (3′ RACE nested primer) (Supplementary Table S1). The predicated coding sequence of *SmPPS1* was PCR-amplified using PPS1-F as the forward primer and PPS1-R as the reverse primer (Supplementary Table S2). The forward and reverse primers used for amplification of *SmPPS2* coding sequence are PPS2-F and PPS2-R, respectively (Supplementary Table S2). Full length cDNA sequences of *SmPPS1* and *SmPPS2* were assembled using Vector NTI 7.1 (Lau et al., 2005).

### Sequence Feature Analysis, Amino Acid Sequence Alignment and Phylogenetic Tree Construction

Sequence feature analysis was performed as described previously (Ma et al., 2012). The amino acid sequences of solanesyl diphosphate synthases from *Arabidopsis* (At2g34630, At1g17050 and At1g78510), rice (OsSPS1 and OsSPS2), *H. brasiliensis* (HbSDS), and *S. miltiorrhiza* (SmPPS1 and SmPPS2) were aligned using Clustal W algorithm-based AlignX, one of the tools of Vector NTI 7.1 software (Thompson et al., 1994; Lau et al., 2005), and then edited by GeneDoc software (Nicholas et al., 1997). For phylogenetic analysis, amino acid sequences of various known prenyltransferases were retrieved from GenBank. Plastid- and mitochondrion-targeting peptides were removed. The maximum likelihood tree was generated using the MABL tool^2^ with default parameters of the “one click” mode (Dereeper et al., 2008). The tree was displayed in the cladogram style with branch length ignored.

### Quantitative Real-Time RT-PCR

Total RNAs isolated from various tissues of *S. miltiorrhiza* were reverse-transcribed into cDNAs using SuperScript III reverse transcriptase (Invitrogen, United States). Quantitative real-time RT-PCR was carried out in a 20 μL volume containing 6 μL diluted cDNA, 0.5 μM forward primer, 0.5 μM reverse primer and 1 × SYBR premix Ex Taq II (Tli RNAseH Plus) (TaKaRa Bio). PCR was performed using the Bio-Rad CFX96 system (Bio-Rad, Hercules, CA, United States) under the following conditions: predenaturation at 95°C for 30 s, annealing at 60°C for 30 s, extension at 72°C for 20 s, 39 cycles, and hold at 4°C. Three biological and three technical replicates were carried out. *SmUBQ10* was used as a reference gene as described previously (Ma et al., 2012). The forward and reverse primers used for *SmPPS1* gene expression analysis are PPS1-Fq and PPS1-Rq, respectively (Supplementary Table S3). For *SmPPS2*, the forward and reverse primers are PPS2-Fq and PPS2-Rq, respectively (Supplementary Table S3). Relative abundance of transcripts was quantified using the comparative CT method as described previously (Livak and Schmittgen, 2008). Standard derivation was calculated from three independent biological replicates. SPSS version 21.0 was used to analyze the variance (ANOVA). *P* < 0.05 and *P* < 0.01 were considered as statistically significant and extremely significant, respectively.

### Functional Complementation of Yeast *coq1* Mutant

The open reading frames (ORFs) of *SmPPS1* and *SmPPS2* were inserted into the yeast expression vector pYES2-CT (Invitrogen, United States) using two restriction enzymes to give the final vectors pYES2-*SmPPS1* (BamH I/Xba I) and pYES2-*SmPPS2* (Kpn I/Not I). The empty vector pYES2-CT and the resulting constructs were separately introduced into the *S. cerevisiae coq1* mutant (strain 3138) using the yeast marker transformation kit (TaKaRa Bio, Japan). Functional complementation assay was performed as described previously (Ducluzeau et al., 2012). The yeast *coq1* mutant with empty vector pYES2-CT and the wild type yeast strain BY4741 were used as controls.

### Determination of UQ and PQ

Yeast cultivation, UQ induction and examination were carried out as described by Ducluzeau et al. (2012) with modifications. Three extractions were performed from each biological replicate. At least three independent biological replicates were carried out. Yeast cells were quantified by absorbance at 600 nm. Cells in 5 mL yeast solution were collected by centrifugation and then washed twice using 2 mL water. One milliliter water containing approximately 500 μL glass beads (0.5 mm) was added to the sediment and then vortexed for 10 min to destroy yeast cell wall. Samples were then quickly mixed with 2 mL acetone and extracted at room temperature for 30 min. The supernatant was evaporated to dryness in a vacuum desiccator at 40°C and re-suspended in 500 μL ethanol. For analysis of UQ in *S. miltiorrhiza*, leaves from 5-week-old *in vitro* cultivated plantlets were grinded into powder in liquid nitrogen and then extracted as described previously with slight modifications (Ohara et al., 2006). Leaf powder (100 mg) was quickly transferred into a 10 mL test tube with 5 mL ethanol, vortexed for 10 s, and then shaken at 120 rpm for 1 h at room temperature. After centrifuged at 10000 rpm for 15 min at 4°C, the supernatant was transferred to a new test tube and the solvent was evaporated in a vacuum desiccator at 40°C. The residue re-suspended in 500 μL of spectroscopically pure ethanol and then filtered through a Bond Elut-C18 solid phase extraction (SPE) column (Agilent, United States) and a 0.22 μm Millipore Express PES membrane filter (Merk Millipore, United States). The filtrate (10 μL) was injected into a C18 reverse-phase column (4.6 × 250 mm, Agilent, United States) and analyzed by HPLC-UV under the following conditions. Solvent system: ethanol/methanol (75:25); flow rate: 1.0 mL/min; temperature: 25°C; detection: absorbance measured at 275 nm. For analysis of PQ in *S. miltiorrhiza*, leaf powder was extracted with cold ethyl acetate as described by Kim et al. (2017) and Zbierzak et al. (2010). After centrifuged at 10000 rpm for 15 min at 4°C, UQ-6 (100 ng, Sigma) was added as internal standard (Zbierzak et al., 2010). The combined mixture was evaporated to dryness in a vacuum desiccator at 40°C and the residue was dissolved in 500 μL ethanol. HPLC-UV analysis was carried out using the isocratic solvent system consisting of ethanol/acetonitrile (1:3) at a flow rate of 1.5 mL/min for 26 min (Hunter et al., 2018). PQ-9 was quantified by comparison with the internal standard and detected spectrophotometrically at 255 nm (Zbierzak et al., 2010; Kim et al., 2017).

### Subcellular Localization of SmPPS1 and SmPPS2

The ORFs of *SmPPS1* and *SmPPS2* without termination codon were PCR-amplified and inserted into pMD-18T (TaKaRa Bio, Japan) to yield T-SmPPS1 and T-SmPPS2 vectors. Sequence-verified clones were digested, gel-purified, and inserted into pCAMBIA1302-GFP, which had a cauliflower mosaic virus 35S promotor upstream of the cloning site. The resulting vectors, 1302-SmPPS1:GFP and 1302-SmPPS2:GFP (Figure 3A), were transformed into *Agrobacterium tumefaciens* strain EHA105 and then injected into leaf lamina of *Nicotiana benthamiana* plants according to the protocol described by Sparkes et al. (2006). After injection for 2–3 days, GFP fluorescence in *N. benthamiana* cells was observed under an Olympus FV3000 confocal microscope (Olympus, Japan) with an excitation filter of 488 nm from the argon laser. Wavelength of 488 nm was also used for fluorescence excitation of chlorophyll. MitoTracker Red CMXRos was used to stain the mitochondria with an excitation filter of 579 nm from the argon laser. Fluorescence emission were collected from 500 to 530 nm for GFP, 650 to 750 nm for chlorophyll, and 590 to 620 nm for MitoTracker Red CMXRos, respectively.

### Binary Vectors Construction and Plant Transformation

The *SmPPS1* and *SmPPS2* coding sequences were cloned and inserted into the binary vector pCAMBIA-1301 to make overexpression constructs. For knockdown of *SmPPS1* and *SmPPS2*, two artificial microRNAs (amiRNAs), termed *amiRPPS1* (5′-TTCTCACTGATGCCATTGTCC-3′) and *amiRPPS2* (5’-TAGTACTCCATGCTGCAGCGA-3’), were designed as described by Schwab et al. (2006) and Shi et al. (2010). To construct knockdown vectors 1301-amiRPPS1 and 1301-amiRPPS2, *P. trichocarpa* ptc-miR408 and ptc-miR408^*^ in a 0.9 kb cDNA of ptc-MIR408 gene were replaced with the designed amiRNA and amiRNA^*^ through overlapping PCR. The resulting sequences were inserted into pCAMBIA1301 (Figures 5A, 7C). The constructs were introduced into *A. tumefaciens* strain GV3101 using the freeze-thaw method (Hofgen and Willmitzer, 1988). *Agrobacterium*-mediated transformation of *S. miltiorrhiza* was performed as described previously (Wang M. et al., 2017). Transgenic shoots were regenerated and selected on 1/2 Murashige-Skoog agar media supplemented with hygromycin 30 mg/L for about 20 days, and then excised and transferred to new 1/2 Murashige-Skoog agar media till rooting. Plantlets with well-developed leaves and roots were propagated. Transgenics were verified by PCR analysis of genomic DNA using *HPTII* gene specific primers, 5′-AGCCTGAACTCACCGCGACG-3′ and 5′-TTGCCCTCGGACGAG TGCTG-3′.

### Chlorophyll, Carotenoid and Malondialdehyde (MDA) Content Analyses

Chlorophylls and carotenoids were extracted from the leaves of 5-week-old *in vitro* cultivated *S. miltiorrhiza* plants as described previously (Xing et al., 2010). Three extractions were carried out from each biological replicate. Light absorbance (A) of the final solution was measured at 663, 647, and 470 nm. The concentrations of chlorophyll a (Chla), chlorophyll b (Chlb) and carotenoids (Car) were calculated as described (Lichtenthaler, 1987). MDA content was determined using the thiobarbituric acid method with slight modifications (Dhindsa and Matowe, 1981). Leaves (0.2 g) from 5-week-old *in vitro* cultivated *S. miltiorrhiza* plants were homogenized in 3 mL 10% thiobarbituric acid and then stood at 4°C overnight. Supernatant was collected after centrifuged at 5000 rpm for 10 min at 4°C. Mixed 2 mL supernatant and 2 mL 0.6% thiobarbituric acid, immediately reacted in boiling water for 15 min, and then terminated by cooling down. The reaction mixture was centrifuged at 5000 rpm for 10 min at 4°C. Light absorbance (A) of the supernatant was measured at 450, 532, and 600 nm. The content of MDA was calculated as follows (Fan et al., 2012): MDA (μmol/L) = 6.456 × (OD_532_ – OD_600_) – 0.566 × OD_450_.

### Transmission Electron Microscopy (TEM) and Scanning Electron Microscopy (SEM) Assays

Leaves harvested from 5-week-old wild type (WT) and amiRPPS1 transgenic plants were fixed in 2.5% glutaraldehyde for 1 week at 4°C. Tissue samples were rinsed three times in PBS buffer, soaked in 1% osmium tetroxide at room temperature for overnight, and dehydrated in a graded ethanol series. The samples were then embedded in Spurr resin for overnight and polymerized at 65°C for 2 days. Uranyl sections (70 nm) were cut with a Leica EM UC6 ultramicrotome (Leica, Germany), stained with 4.5% uranyl acetate for 5 min and lead citrate for 2 min, and then observed under a JEM-1400 TEM (JEOL, Japan).

Scanning electron microscopy analysis of leaves from 5-week-old WT and amiRPPS1 transgenic lines were carried out following the procedure of Irish and Sussex (1990) with some modifications. Leave samples were fixed in 2.5% glutaraldehyde overnight at 4°C, dehydrated in a graded ethanol series, and then immersed in tert-butanol for 30 min. The samples were dried in the JFD-320 Freeze Drying Device (JEOL, Japan), coated with gold dust in JFC-1600 auto fine coater (JEOL, Japan), and then placed on SEM stubs for examination under a JSM-6510LV SEM (JEOL, Japan).

## Results

### Identification of Two *PPS* Genes in *S. miltiorrhiza*

Through BLAST analysis of previously known plant solanesyl diphosphate synthase sequences against the current assembly of *S. miltiorrhiza* (line 99–3) genome (Altschul et al., 1990, 1997; Xu et al., 2016), we identified two putative *S. miltiorrhiza* long-chain PPS genes, termed *SmPPS1* and *SmPPS2*, respectively. In order to verify computational prediction and obtain full length cDNA sequences, 5′ RACE, 3′ RACE and RT-PCR were performed. The results showed that *SmPPS1* had a 5′-untranslated region (UTR) of 210 bp, a 3′-UTR of 300 bp, and an ORF of 1266 bp. The length of 5′- and 3′-UTRs of *SmPPS2* was 155 and 125 bp, respectively, and its ORF was 1275 bp. BLAST analysis of SmPPS1 and SmPPS2 against the GenBank nucleotide database showed that *SmPPS1* had the highest homology (80%) with *H. brasiliensis HbSDS* (Phatthiya et al., 2007). The deduced amino acid sequence of *SmPPS1* showed 79% identity to *Arabidopsis* SPS isoform At1g78510. The results verify that SmPPS1 is a homolog of plant SPSs. The coding sequence of the cloned *SmPPS2* cDNA showed four nucleotide differences with the predicted coding sequence of *SmGPPS* (GenBank accession number JN831107.1) (Ma et al., 2012). However, their amino acid sequences are identical. It suggests that *SmPPS2* is full length cDNA of the previously predicted *SmGPPS* (Ma et al., 2012). Four nucleotide differences between *SmPPS2* and *SmGPPS* may be caused by sequencing errors or single nucleotide polymorphisms. The deduced SmPPS2 protein sequence showed 70–83% identities with known SPSs in plant species, such as *Sesamum indicum*, *Erythranthe guttata, Nicotiana attenuata*, and 70–80% identities with homodimeric geranyl diphosphate synthases in various angiosperm plants, such as *S. lycopersicum, Catharanthus roseus*, *Citrus sinensis*, and *Arabidopsis* (Ma et al., 2012). The similarity between SPSs and GPPSs could result in erroneous annotation of gene function (Ma et al., 2012). Full length cDNA sequence of *SmPPS1* and *SmPPS2* has been deposited to GenBank under the accession numbers of MH924998 and JX090100, respectively.

### Sequence Features, Conserved Domains and Phylogenetic Relationships of *SmPPSs*

**FIGURE 1:**
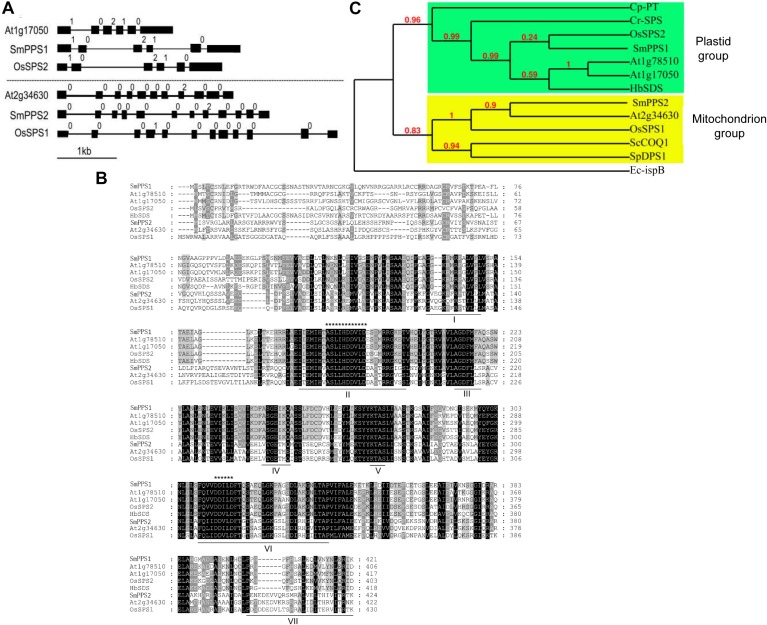
Comparative analysis of *SmPPS1* and *SmPPS2* in *S. miltiorrhiza* and various other plants. **(A)** Structure organization of *SmPPS1* and *SmPPS2* from *S. miltiorrhiza*, *Arabidopsis* and Rice. The exon/intron structures of coding regions were analyzed on the GSDS webserver (http://gsds.cbi.pku.edu.cn/) (Hu et al., 2015). Exons are represented by solid black boxes, introns are represented by lines between the boxes, and intron phases are indicated by 0, 1, and 2 (Long et al., 1995). GenBank accession numbers for full length *SmPPS1* and *SmPPS2* genes are JQ763411 and JN831107, respectively. The gene ID of *OsSPS1* and *OsSPS2* are AK071299 and AK066579, respectively. Different groups of *SPSs* are divided by dashed lines. **(B)** Amino acid sequence alignment of long-chain polyprenyl diphosphate synthases from various plant species. Seven domains conserved in eukaryotic polyprenyl diphosphate synthases are underlined and indicated by “I” to “VII”. The FARM signature and the SARM signature are indicated by “^*************^” and “^******^”, respectively. **(C)** Phylogenetic relationships of long-chain prenyltransferases from various organisms. The maximum likelihood tree was generated using the MABL tool (http://www.phylogeny.fr/). The accession numbers are listed as follows. SmPPS1, *S. miltiorrhiza* (MH924998); SmPPS2, *S. miltiorrhiza* (JX090100); At1g17050, *A. thaliana* (BT028962); At1g78510, *A. thaliana* (BT025550); At2g34630, *A. thaliana* (BT020524); HbSDS, *Hevea brasiliensis* (DQ437520); Cr-SPS, *Chlamydomonas reinhardtii* (XM_001693378.1); Cp-PT, *Cyanophora paradoxa* (NC_001675.1); ScCOQ1, *S. cerevisiae* (J05547); SpDPS1, *S. pombe* (D84311); Ec-ispB, *E. coli* (NP_417654.1); OsSPS1, *O. sativa* (AK071299); OsSPS2, *O. sativa* (AK066579).

Sequence comparison of the two *SmPPS* genes and their full length cDNAs showed that *SmPPS1* contained 5 introns, whereas the number of introns in *SmPPS2* was 11. It suggests the complexity of *SmPPS2* in gene structure. Analysis of the exon/intron structures of *SmPPS1*, *SmPPS2* and the *SPSs* from *Arabidopsis* and rice showed that the long-chain prenyltransferase genes could be divided into two distinct groups, each of which had similar intron/exon structures (Figure 1A). *SmPPS1* belongs to the group of *SPSs* (At1g17050, At1g78510, and OsSPS2) involved in the biosynthesis of PQ side chain in the chloroplasts, whereas *SmPPS2* is a member of the group of *SPSs* (At2g34630 and OsSPS1) involved in the biosynthesis of UQ side chain in the mitochondria. The first group of *PPSs* contains six exons and five introns, and has the same intron phases (the position of an intron within or between codons). The exon/intron structures of the second group of *PPSs* are relatively complicated (Figure 1A).

Conserved domain analysis against the GenBank CDD database showed that SmPPS1 and SmPPS2 proteins contained the conserved Trans_IPPS_HT domain of trans-type long-chain prenyltransferases (Marchler-Bauer et al., 2011). Sequence alignment of SmPPS1, SmPPS2 and other plant SPS proteins showed the existence of seven conserved domains previously found in SPSs from various living organisms (Figure 1B) (Saiki et al., 2003; Jun et al., 2004). FARM (the first aspartate-rich motif, DDxxD) and SARM (the second aspartate-rich motif, DDxxD) are two of the seven conserved domains. They exist in all plant SPSs and are putatively involved in binding prenyl diphosphate substrates (Wang and Ohnuma, 1999). SmPPS1 shares the same ASLIHDDVLD “FARM signature” with plastidial SPSs, such as At1g17050 and OsSPS2, whereas SmPPS2 has the same ASLLHDDVLD “FARM signature” with mitochondrial SPSs, such as At2g34630 and OsSPS1 (Figure 1B). Consistently, protein subcellular localization prediction on the TargetP 1.1 webserver^3^ showed that SmPPS1 was localized in the chloroplasts, whereas SmPPS2 was localized in the mitochondria. It suggests that SmPPS1 and SmPPS2 may be functionally distinct.

Phylogenetic analysis showed that plant *trans*-long-chain prenyltransferases could be divided into two different subgroups, including the plastid subgroup and the mitochondrion subgroup (Figure 1C). SmPPS1 belongs to the plastid subgroup, which includes PQ biosynthetic prenyltransferases, such as *Arabidopsis* At1g78510, At1g17050, rice OsSPS2, and other SPSs exclusively from oxygenic photosynthetic organisms (Figure 1C). SmPPS2 belongs to the mitochondrion subgroup, which includes UQ biosynthetic prenyltransferases, such as At2g34630, OsSPS1, and yeast COQ1 (Figure 1C) (Ohara et al., 2010; Ducluzeau et al., 2012). The results showed that SmPPS1 and SmPPS2 could be involved in the biosynthesis of PQ in the chloroplasts and UQ in the mitochondria, respectively.

### Differential and Light-Induced Expression of *SmPPS1* and *SmPPS2*

**FIGURE 2:**
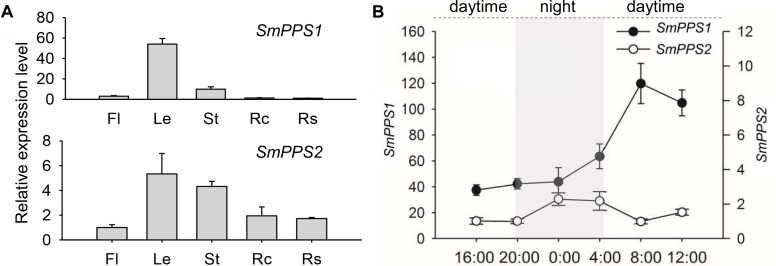
Expression of *SmPPS1* and *SmPPS2* in *S. miltiorrhiza*. **(A)** Tissue-specific expression of *SmPPS1* and *SmPPS2* in flowers (Fl), leaves (Le), stems (St), root cortices (Rc), and root steles (Rs). Relative abundances of *SmPPS1* and *SmPPS2* in Rs and Fl are arbitrarily set to 1. **(B)** Different expression patterns of *SmPPS1* and *SmPPS2* in response to light induction in *S. miltiorrhiza* leaves. Light gray and white represent night and daytime, respectively.

*SmPPS2* has been previously shown to be ubiquitously expressed, although its expression in leaves and stems is slightly higher than in flowers, root cortices and root steles of 2-year-old, field-grown *S. miltiorrhiza* (Ma et al., 2012). In this study, we analyzed the expression of *SmPPS1* in the same tissues used for the analysis of *SmPPS2*. The results showed that *SmPPS1* was highly expressed in leaves, followed by stems (Figure 2A). The expression of *SmPPS1* in flowers, root cortices and root steles was very low. It suggests *SmPPS1* predominantly accumulates in green tissues. The results are consistent with the predicted functions of *SmPPS1* in PQ biosynthesis in the chloroplasts and *SmPPS2* in UQ biosynthesis in the mitochondria.

The expression patterns of *SmPPS1* and *SmPPS2* were analyzed on a summer day in Beijing. The sun rises at about 4:45 and sets at about 20:00, so the time for daytime and night is about 15 and 9 h, respectively. As a result, the transcription of *SmPPS1* and *SmPPS2* were induced in day/night circulation with different expression patterns (Figure 2B). When daytime appeared, the transcript level of *SmPPS1* began to accumulate rapidly and peaked at eight o’clock in the morning and then slowly decreased to a relatively stable level. The level of *SmPPS2* decreased slightly during daytime and increased slowly at 20:00 when night began. *SmPPS2* showed a relatively high expression level from midnight (zero o’clock) to four o’clock. Different expression patterns of *SmPPS1* and *SmPPS2* in response to light induction conformed to their predicated functions in the biosynthesis of PQ and UQ, respectively, which are two electron transporters in photophosphorylation and oxidative phosphorylation (Liu and Lu, 2016).

### Subcellular Localization of SmPPS1 and SmPPS2

**FIGURE 3:**
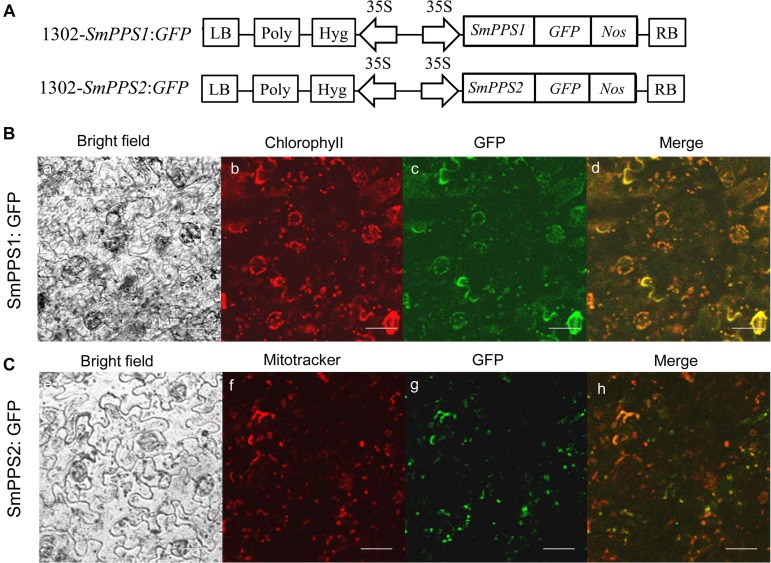
Subcellular localization of SmPPS1 and SmPPS2 fused to GFP proteins in *N. benthamiana*. **(A)** Schematic representation of T-DNA in 1302-*SmPPS1*:*GFP* and 1302-*SmPPS2*:*GFP* plasmids. LB, left boundary of T-DNA; Poly, poly(A) signal; *Hyg*, hygromycin phosphotranferase gene; *35S*, the promoter of CaMV 35S; *GFP*, green fluorescent protein gene; *Nos*, the terminator of nopaline synthase gene; RB, right boundary of T-DNA. **(B)** Transient expression of *SmPPS1*:*GFP* in *N. benthamiana* leaves. **(C)** Transient expression of *SmPPS2*:*GFP* in *N. benthamiana* leaves. Bright field, bright field channel; Chlorophyll, red pseudocolor of plastid auto-fluorescence; GFP, green pseudocolor of SmPPS1:GFP and SmPPS2:GFP; Mitotracker, red pseudocolor of Mitotracker; Merge, the merged image.

Prediction of protein subcellular localization using TargetP 1.1 showed that SmPPS1 and SmPPS2 could be localized in the chloroplasts and the mitochondria, respectively. In order to experimentally test the localization of SmPPS1 and SmPPS2 *in planta*, full length SmPPS1 and SmPPS2 were in-frame fused with a green fluorescent protein gene (GFP). The structures of the two resulting expression vectors, termed 1302-SmPPS1:GFP and 1302-SmPPS2:GFP, were shown in Figure 3A. Fusion genes were transformed into *N. benthamiana* leaves and transiently expressed. Observation of the transformed cells using a confocal laser scanning microscope showed that green fluorescence from SmPPS1:GFP completely coincided with red dots from chlorophyll autofluorescence (Figure 3B). It suggests that SmPPS1 was localized in the chloroplasts. Different from SmPPS1, SmPPS2:GFP showed a more complex localization. Most of the green fluorescence from SmPPS2:GFP showed a similar pattern to the Mitotracker red fluorescence in the mitochondria, whereas some of the other green fluorescence signals dissociated with the mitochondria. It indicated that SmPPS2 was, but not solely, localized in the mitochondria (Figure 3C).

### Yeast Complementation of *SmPPS1* and *SmPPS2*

**FIGURE 4:**
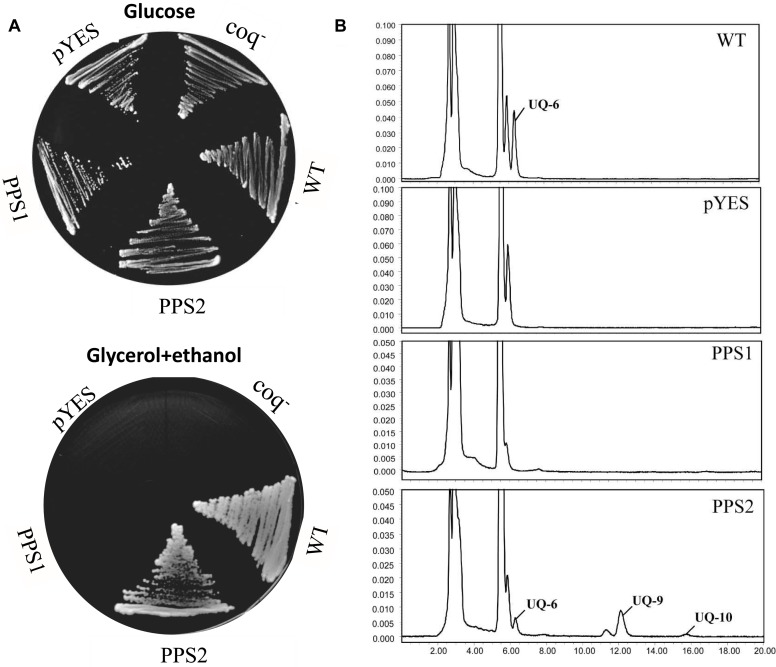
Yeast complementation assays of *SmPPS1* and *SmPPS2*. **(A)** *SmPPS2* functionally complemented the yeast *coq1* knockout mutant. Yeast cells were plated on an YPD medium with glucose as the carbon source, or an YPEG medium with glycerol and ethanol as the carbon sources and 0.05% D-galactose as the inducer. **(B)** HPLC analysis of UQ in WT yeast and *coq1* mutant harboring pYES2-CT, pYES2-*SmPPS1*, and pYES2-*SmPPS2*, respectively. pYES, yeast *coq1* knockout strain 3138 harboring empty vector pYES2-CT; PPS1, yeast *coq1* knockout strain 3138 harboring pYES2-*SmPPS1*; PPS2, yeast *coq1* knockout strain 3138 harboring pYES2-*SmPPS2*; WT, wild type parent yeast strain BY4741; coq^–^, yeast coq1 knockout strain 3138.

It has been showed that the *coq1* mutant of *S. cerevisiae* (3138) is unable to produce UQ and cannot grow on the minimum medium containing non-fermentable carbon source, since it lacks the mitochondrial hexaprenyl diphosphate synthase activity (Ashby and Edwards, 1990; Gin and Clarke, 2005). In order to further investigate the function of *SmPPSs, SmPPS1* and *SmPPS2* cDNAs were subcloned into the yeast D-galactose-inducible expression vector pYES2-CT to yield pYES2-SmPPS1 and pYES2-SmPPS2, respectively. The resulting constructs were introduced into the *coq1* mutant 3138. As shown in Figure 4A, the expression of *SmPPS2* could restore the ability of mutant cells to use glycerol and ethanol as carbon sources, while no functional rescues were found for 3138 harboring pYES2-SmPPS1 or empty vector pYES2-CT. HPLC analysis showed that 3138 with pYES2-SmPPS2 produced UQ-6, UQ-9 and a small amount of UQ-10, and these UQs were not found in 3138 with pYES2-SmPPS1 or empty vector pYES2-CT (Figure 4B). The results indicate that SmPPS2 is a functional prenyltransferases involved in UQ-9 and UQ-10 production in *S. miltiorrhiza*. In addition to the peaks of UQ-6, UQ-9, and UQ-10, we noticed that there was a peak between the peaks of UQ-6 and UQ-9 in 3138 with pYES2-SmPPS2, and the peak in front of the peak of UQ-6 was changed significantly in different yeast cells analyzed. The compounds for these peaks remain to be identified.

### Functional Characterization of *SmPPS1* in Transgenic *S. miltiorrhiza* Plants

**FIGURE 5:**
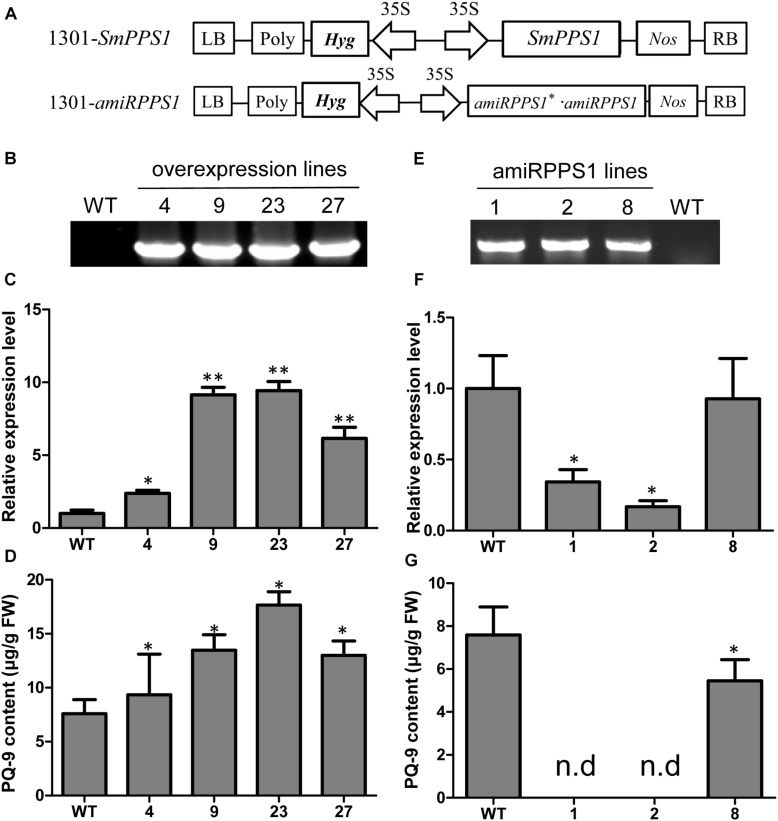
*SmPPS1* expression level and PQ-9 content in *SmPPS1* overexpression and knockdown transgenic *S. miltiorrhiza* lines. **(A)** Schematic representation of T-DNA in 1301-*SmPPS1* and 1301-amiR*PPS1* plasmids. LB, left boundary of T-DNA; Poly, poly(A) signal; *Hyg*, hygromycin phosphotranferase gene; 35S, the promoter of CaMV 35S; *GFP*, green fluorescent protein gene; *Nos*, the terminator of nopaline synthase gene; RB, right boundary of T-DNA. **(B)** PCR verification of WT and *SmPPS1* overexpression lines. **(C)** Relative expression level of *SmPPS1* in WT and *SmPPS1* overexpression lines. **(D)** PQ-9 content in WT and *SmPPS1* overexpression lines. **(E)** PCR verification of WT and *SmPPS1* knockdown lines. **(F)** Relative transcript level of *SmPPS1* in WT and *SmPPS1* knockdown lines. **(G)** PQ-9 content in WT and *SmPPS1* knockdown lines. WT, wild type; 4, 9, 23, and 27, *SmPPS1* overexpression lines PPS1ox-4, PPS1ox-9, PPS1ox-23, and PPS1ox-29; 1, 2 and 8, *SmPPS1* knockdown lines amiRPPS1-1, amiRPPS1-2 and amiRPPS1-8; n.d, not detected. *P* < 0.05 (^*^) and *P* < 0.01 (^∗∗^) were considered statistically significant and extremely significant, respectively.

Further functional analysis of *SmPPS1* was carried out using *SmPPS1* transformed *S. miltiorrhiza* plants. The transgenics were obtained using the *Agrobacterium*-mediated transformation method (Wang M. et al., 2017). A total of four *SmPPS1* overexpression lines, termed PPS1ox-4, PPS1ox-9, PPS1ox-23 and PPS1ox-29, respectively, were analyzed (Figure 5B). qRT-PCR showed that, among the four analyzed lines, PPS1ox-23 had the highest *SmPPS1* expression level, which was about 9.4 times the level of *SmPPS1* in WT plants (Figure 5C). Consistently, HPLC-UV analysis showed that PQ-9 content in PPS1ox-23 increased by 2.3 times compared with that in WT (Figure 5D). The lowest *SmPPS1* expression level was detected in PPS1ox-4. It was about 2.3 times of the *SmPPS1* expression level in WT (Figure 5D). PQ-9 content in PPS1ox-4 was 23% higher than that in WT (Figure 5D). The results suggest that *SmPPS1* overexpression can increase PQ-9 content.

**FIGURE 6:**
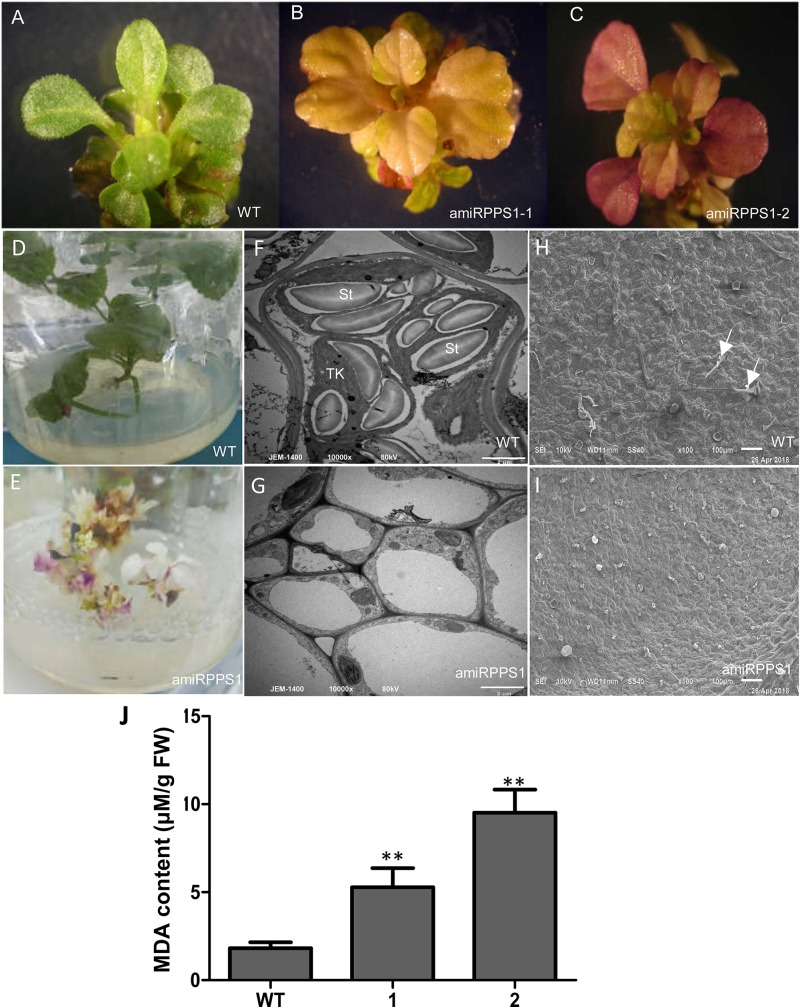
The phenotype and MDA content of amiRPPS1 transgenic *S. miltiorrhiza* lines. **(A)** wild-type plants. **(B)** amiRPPS1-1 plants. **(C)** amiRPPS1-2 plants. **(D,E)** WT **(D)** and amiRPPS1-1 **(E)** plants grown on 1/2 MS medium for 5 weeks. **(F,G)** TEM analysis of chloroplast development in leaf cells of WT **(F)** and amiRPPS1-1 **(G)**. St, starch granule; TK, thylakoid. **(H,I)** SEM analysis of trichome development in WT **(H)** and amiRPPS1-1 **(I)**. Arrows indicate trichomes. **(J)** MDA content in WT and amiRPPS1 lines. WT, wild type; 1, amiRPPS1-1; 2, amiRPPS1-2. “^∗∗^” means statistically extremely significant at *P* < 0.01.

In order to down-regulate *SmPPS1* expression in *S. miltiorrhiza*, the artificial microRNA method was used. Three *SmPPS1* knockdown lines, named amiRPPS1-1, amiRPPS1-2 and amiRPPS1-8, respectively, were analyzed (Figure 5E). Among them, lines amiRPPS1-1 and amiRPPS1-2 showed significant decrease in *SmPPS1* expression (Figure 5F). Consistently, PQ-9 content in these transgenic lines was reduced to below the detection limit (Figure 5G). AmiRPPS1-1 plants exhibited albino and dwarf phenotypes (Figures 6D,E), and leaf color might change to yellow during cultivation (Figures 6A,B). Further determination of chlorophyll and carotenoid contents showed that, compared with WT, the content of chlorophylls and carotenoids in amiRPPS1-1 leaves were dramatically reduced (Table 1). Chlorophyll a content decreased to 0.92% of WT, chlorophyll b reduced to 0.27%, carotenoids decreased to about 0.79%, and total chlorophylls decreased to about 0.13%. In addition, the ratio of chlorophyll a and chlorophyll b also decreased (Table 1). TEM observation of amiRPPS1-1 leaves showed that silence of *SmPPS1* in *S. miltiorrhiza* resulted in abnormal development of chloroplasts. The chloroplasts in amiRPPS1-1 leaves lacked starch granules and thylakoids, and were smaller than the chloroplasts in WT (Figures 6F,G). SEM analysis showed that, compared to the WT, the development of trichomes was affected seriously in amiRPPS1-1 plants (Figures 6H,I). It seems that trichome development was blocked at very early developmental stage. AmiRPPS1-2, another *SmPPS1* knockdown line with PQ-9 level below detection limit (Figure 5F), showed yellow and deep orange coloration on their leaves (Figure 6C). Since abnormal development of chloroplasts and color changes in amiRPPS1 transgenic lines, we speculated that the plants were undergoing oxidative stress. To test this hypothesis, the content of MDA, a reactive aldehyde and a biomarker of oxidative stress, was measured. The results showed significant increase of MDA content in amiRPPS1-1 and amiRPPS1-2 plants (Figure 6J), which confirmed the speculation. Taken together, knockdown of *SmPPS1* gene may affect the biosynthesis of chlorophylls and carotenoids and the development of chloroplasts and trichomes, and cause toxic stress in cells.

**TABLE 1:** The content of chlorophylls and carotenoids in WT and amiRPPS1-1.

	**Chla (mg/g)**	**Chlb(mg/g)**	**Car(mg/g)**	**Chl(mg/g)**	**Chla/b**
WT	50 ± 5.1	14 ± 1.6	13 ± 1.2	64 ± 6.6	3.62 ± 0.11
amiRPPS1-1	0.46 ± 0.23^∗∗^	0.38 ± 0.25^∗∗^	1.03 ± 0.3^∗∗^	0.84 ± 0.3^∗∗^	1.2 ± 0.17^*^

Leaves from 5-week-old plants were harvested for determination of chlorophyll and carotenoid contents. Differences of the means were compared between WT and amiRPPS1-1. Chla, chlorophyll a; Chlb, chlorophyll b; Car, carotenoid; Chl, total chlorophyll; Chla/b, chlorophyll a/chlorophyll b. *P* < 0.05 (^*^) and *P* < 0.01 (^∗∗^) were considered statistically significant and extremely significant, respectively.

### Determination of UQ in *S. miltiorrhiza* and Functional Characterization of *SmPPS2* in Transgenic Plants

**FIGURE 7:**
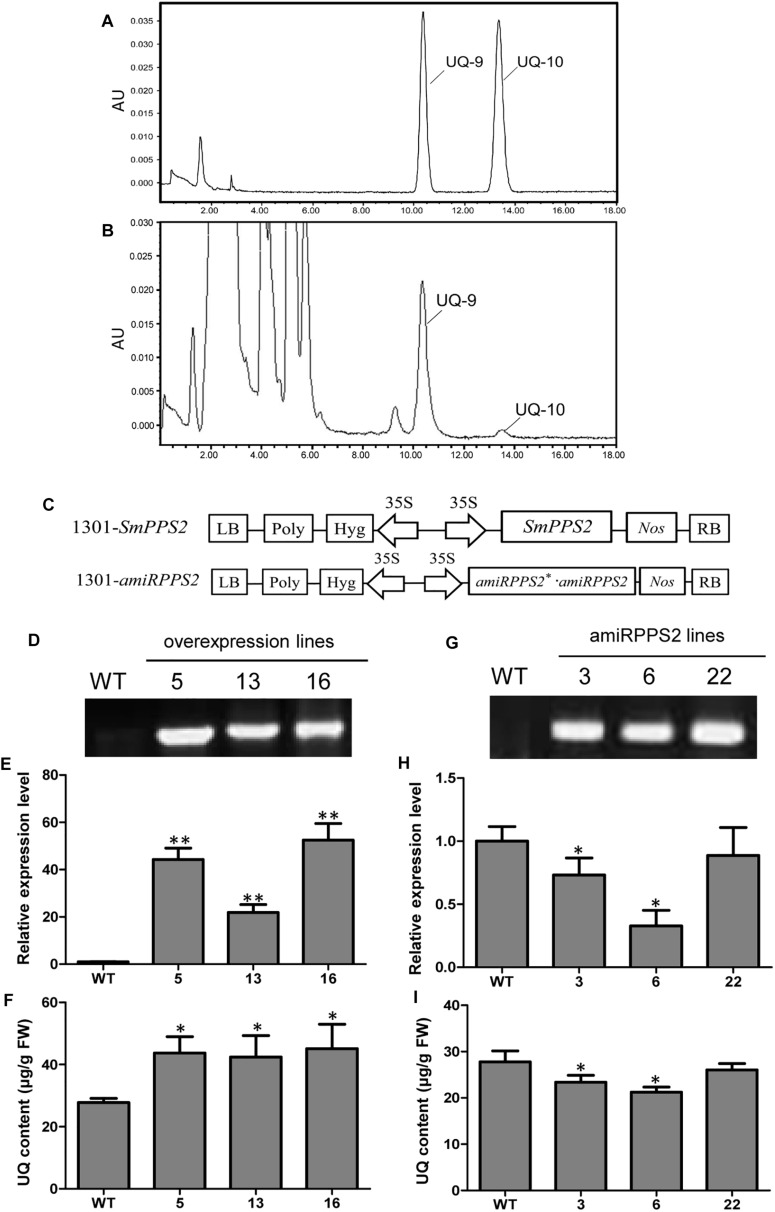
Analysis of UQ in wild-type and *SmPPS2* overexpression and knockdown *S. miltiorrhiza* plantlets. **(A)** Separation of UQ-9 and UQ-10 standards; **(B)** Analysis of UQ-9 and UQ-10 in *S. miltiorrhiza* leaves. **(C)** Schematic representation of T-DNA in 1301-*SmPPS2* and 1301-amiR*PPS2* plasmids. LB, left boundary of T-DNA; Poly, poly(A) signal; *Hyg*, hygromycin phosphotranferase gene; 35S, the promoter of CaMV 35S; *GFP*, green fluorescent protein gene; *Nos*, the terminator of nopaline synthase gene; RB, right boundary of T-DNA. **(D)** PCR verification of WT and *SmPPS2* overexpression lines. **(E)** Relative expression level of *SmPPS2* in WT and *SmPPS2* overexpression lines. **(F)** UQ content in WT and *SmPPS2* overexpression lines. **(G)** PCR verification of WT and *SmPPS2* knockdown lines. **(H)** Relative expression level of *SmPPS2* in WT and *SmPPS2* knockdown lines. **(I)** UQ content in WT and *SmPPS2* knockdown lines. WT, wild type; 5, 13 and 16, *SmPPS2* overexpression lines PPS2ox-5, PPS2ox-13 and PPS2ox-16; 3, 6 and 22, *SmPPS2* knockdown lines amiRPPS2-3, amiRPPS2-6 and amiRPPS2-22. *P* < 0.05 (^*^) and *P* < 0.01 (^∗∗^) were considered statistically significant and extremely significant, respectively.

Yeast complementation assay showed that *SmPPS2* could functionally complement the *coq1* mutation and catalyzed the production of UQ-9 and UQ-10 in yeast cells (Figure 4). To verify the function of *SmPPS2* in *S. miltiorrhiza*, we analyzed the type and content of UQ in roots, stems and leaves of *S. miltiorrhiza* using HPLC-UV. The result showed that both UQ-9 and UQ-10 were produced in *S. miltiorrhiza* plants with the greatest content in leaves [27.97 ± 6.58 μg/g fresh weight (FW)], less in stems (8.85 ± 0.55 μg/g FW), and the least in roots (3.62 ± 1.13 μg/g FW) (Figures 7A,B and Table 2). The content of UQ-10 varies from 0.46 ± 0.29 μg/g FW in stems to 0.31 ± 0.04 μg/g FW in roots, which showed a gentle change compared with UQ-9 content varying significantly between 27.4 ± 6.58 μg/g FW in leaves and 3.32 ± 1.78 μg/g FW in roots. It is consistent with the results from yeast complementation assay.

**TABLE 2:** UQ content in roots, stems and leaves of *S. miltiorrhiza*.

**Tissue**	**UQ content (μg/g FW)**		
	**UQ-9**	**UQ-10**	**Total UQ**
Root	3.32 ± 1.78	0.31 ± 0.04	3.62 ± 1.13
Stem	8.39 ± 0.52	0.46 ± 0.29	8.85 ± 0.55
Leaf	27.4 ± 6.58	0.45 ± 0.01	27.97 ± 6.58

*The means ± SD of three replicates are shown. FW, fresh weight.*

To further analyze the function of *SmPPS2* in plants, transgenic *SmPPS2* overexpression and artificial miRNA lines were produced (Figures 7D,G). No visible phenotype changes were observed for these transgenic lines. Quantitative real-time PCR analysis of *SmPPS2* overexpression lines PPS2ox-5, PPS2ox-13, and PPS2ox-16 showed that the levels of *SmPPS2* transcripts were significantly up-regulated from 22 times of WT in PPS2ox-13 to 52.2 times in PPS2ox-16 (Figure 7E). Consistently, HPLC-UV analysis showed that total UQ contents were significantly increased in these *SmPPS2* overexpression lines (Figure 7F). The levels of UQ content in transgenics were from 152 to 163% of WT. It suggests that overexpression of *SmPPS2* can substantially increase UQ production in *S. miltiorrhiza* plants.

In artificial miRNA transgenic *S. miltiorrhiza* lines amiRPPS2-3 and amiRPPS2-6, the levels of *SmPPS2* transcripts were significantly down-regulated (Figure 7H). Consistently, total UQ content was decreased by 7 and 20% of WT in amiRPPS2-3 and amiRPPS2-6, respectively (Figure 7I). Reduction of *SmPPS2* transcripts and total UQ content were also detected in amiRPPS2-22, although the reduction was not statistically significant (Figures 7H,I). These data suggest that alteration of *SmPPS2* expression can affect the accumulation of UQ in *S. miltiorrhiza*.

## Discussion

*Salvia miltiorrhiza* is a widely used traditional Chinese medicine material and an emerging model system for medicinal plant biology (Ma et al., 2012; Song et al., 2013; Deng and Lu, 2017; Zhang and Lu, 2017). So far, more than two hundred chemical compounds have been isolated from this plant (Wang L. et al., 2017; Mei et al., 2019). Lipophilic diterpenoid tanshinones and hydrophilic phenolic acids are two major classes of bioactive compounds in *S. miltiorrhiza* (Gao et al., 2009; Kai et al., 2010; Hao et al., 2013; Xing et al., 2018). Other compounds, such as quinones, flavonoids, anthocyanidins, alkaloids, monoterpenes, sesquiterpenes, triterpenes, sterols and saccharides, were also found in *S. miltiorrhiza* (Wang L. et al., 2017; Mei et al., 2019), although their medicinal value is largely unknown.

Plastoquinone and UQ are two major classes of prenylquinones. In plants, PQ usually contains nine isoprenoid units, whereas the number of isoprenoid units for UQ can be nine or ten, of which UQ-10 is clinically prescribed as a complementary medicine in treating cardiovascular diseases, such as hypertension, hyperlipidemia, coronary artery disease and heart failure (Liu and Lu, 2016). UQ-10 supplementation energizes the body and increases body energy production in the form of ATP. It is recognized as a kind of super vitamin and known as vitamin Q (Shukla and Dubey, 2018). Through HPLC-UV analysis, we reported for the first time that both UQ-9 and UQ-10 were produced in *S. miltiorrhiza*. Although the medicinal significance of UQ-10 in this plant remained to be elucidated, it is possible that UQ-10 may strengthen the therapeutic effect of *S. miltiorrhiza* in clinical practice.

It has been shown that the content of UQ varies significantly in different organs of a plant and in different plant species. In *O. sativa*, UQ-9 content is 8.7 ± 5.0, 9.3 ± 3.7, and 7.7 ± 2.0 μg/g dry weight (DW) in roots, stems and leaves, respectively, whereas it is only 1.4 ± 0.2 μg/g DW in grains (Ohara et al., 2006). Among vegetable oils, maize germ oil and sunflower refined oil contain abundant UQ-9. The content of UQ-9 in maize germ oil and sunflower refined oil is 233.71 and 101.3 μg/g, respectively (Rodríguez-Acuña et al., 2008; Pyo, 2010). In soybean and rapeseed oils, UQ-10 contents are relatively high, which are 92.3 μg/g (Kamei et al., 1986) and 63.5 μg/g (Mattila and Kumpulainen, 2001), respectively. In addition, some other vegetables and fruits also contain UQ-9 and UQ-10. However, the contents are much less compared with those in vegetable oils. For instance, the content of UQ-9 and UQ-10 in pea, cauliflower, apple and strawberry are 0.1 and 1.7 μg/g FW, 0.04 and 2.7 μg/g FW, 0.2 and 1.3 μg/g FW, and 0.1 and 1.4 μg/g FW, respectively (Mattila and Kumpulainen, 2001). In this study, we found that both UQ-9 and UQ-10 were produced in leaves, stems and roots of *S. miltiorrhiza* plants. The highest total UQ content is 27.97 ± 6.58 μg/g FW, which was found in leaves. Generally speaking, the content of total UQ in *S. miltiorrhiza* is higher than that in rice, vegetables and fruits but less than that in vegetable oils (Kamei et al., 1986; Mattila and Kumpulainen, 2001; Ohara et al., 2006; Rodríguez-Acuña et al., 2008; Pyo, 2010).

The pathway and enzyme genes of UQ biosynthesis have been well studied in bacteria and yeasts; however, they are largely unknown in plants (Liu and Lu, 2016). For instance, the current model for UQ biosynthesis in plants was proposed based on the biosynthetic pathway of yeast UQ and has not been experimental demonstrated (Hayashi et al., 2014); the majority of UQ biosynthetic enzyme genes have not been functionally analyzed in plants, although orthologs have been identified from *Arabidopsis* and are able to complement yeast mutants; *Arabidopsis* does not contain the ortholog of *CoQ7*, which encodes a mono-oxygenase involved in the penultimate step of UQ biosynthesis (Hayashi et al., 2014); in addition, a few UQ biosynthetic enzyme genes are still unknown even in yeast cells (Hayashi et al., 2014). Moreover, although PQ biosynthetic pathway is relatively clear, genes involved in PQ biosynthetic pathway have mainly studied in *Arabidopsis*.

Polyprenyl diphosphate synthases are key enzymes to determine the number of isoprenoid units in the biosynthesis of isoprenoid compounds and their derivatives, such as monoterpenes, diterpenes, triterpenoids, PQ, UQ, and vitamin E (Liu and Lu, 2016). Based on the chain length of final products, PPSs can be divided into three classes, including short-, medium- and long-chain PPSs (Hemmi et al., 2002). Among them, long-chain PPSs are significant to PQ and UQ biosynthesis in plants. So far, *PPS* genes have been identified from *Arabidopsis*, rice, tomato and *H. brasiliensis* (Jun et al., 2004; Phatthiya et al., 2007; Ohara et al., 2010; Ducluzeau et al., 2012; Jones et al., 2013). However, functional characterization through transgenic plants has only been performed for *Arabidopsis At2g34630* and tomato *SlSPS* and *SlDPS* (Ducluzeau et al., 2012; Jones et al., 2013). Analysis of *Arabidopsis* At2g34630 T-DNA mutants and overexpressing lines showed that At2g34630 was involved in UQ-9 biosynthesis (Ducluzeau et al., 2012). Through virus-induced gene silencing of *SlSPS* and *SlDPS* and overexpression of tomato *SlSPS* in tobacco, Jones et al. (2013) showed that tomato *SlDPS* is involved in UQ-10, whereas *SlSPS* is involved in PQ-9 biosynthesis. In this study, we identified and characterized two long-chain *SmPPS* genes from *S. miltiorrhiza*. The cloned nucleotide sequence of *SmPPS2* is almost identical to the ORF of *SmGPPS* previously annotated to be involved in terpenoid biosynthesis in *S. miltiorrhiza* (Ma et al., 2012), and the deduced amino acid sequence of SmPPS2 and SmGPPS are identical. It suggests that the cloned *SmPPS2* is full length cDNA of the annotated *SmGPPS*. Similar case of *SmPPS2* was found for *Arabidopsis* At2g34630 (*AtPPPS*), which had been annotated as AtGPPS, but later was proved to be a SPS involved in the biosynthesis of UQ side chain (Hsieh et al., 2011; Ducluzeau et al., 2012). The reason causing erroneous annotation is probably that GPPSs are evolutionarily most closely related to long-chain PPSs in angiosperms (Hsieh et al., 2011). Functional analysis of *SmPPSs* through stable gene overexpression and silencing in the original plant species showed that *SmPPS1*, similar to *SlSPS*, was involved in PQ-9 biosynthesis, whereas *SmPPS2* was involved in the biosynthesis of UQ-9 and UQ-10, with the major product to be UQ-9. It suggests the difference among the function of *SmPPS2*, *SlDPS*, and At2g34630.

In consistent with their functions in photophosphorylation-related PQ and oxidative phosphorylation-related UQ, *SmPPS1* and *SmPPS2* showed differential responses to light induction. When a day begins, plant photosynthesis and photophosphorylation are enhanced with the increase of sunlight. Up-regulation of *SmPPS1* may facilitate PQ production to help the photophosphorylation process. Oxidative phosphorylation in respiration has little relevance to light, so the expression of *SmPPS2* was relatively stable during daytime.

Experimental GFP analysis of *N. benthamiana* leaves showed that the green pseudocolor of SmPPS1:GFP was co-localized with the red pseudocolor of chloroplast autofluorescence. It suggests that SmPPS1 is localized in the chloroplasts. The results are consistent with previous results for At1g78510, *OsSPS2* and *SlSPS* involved in PQ side chain biosynthesis (Ohara et al., 2010; Ducluzeau et al., 2012; Block et al., 2013; Jones et al., 2013). Experimental analysis of SmPPS2:GFP localization showed that the green fluorescence was not only co-localized with the red fluorescence of mitochondria stained with a marker dye MitoTracker, but also found outside of the mitochondria. It suggests that, in addition to the mitochondria, SmPPS2 is probably localized in other organelles. Similarly, it has been shown that At2g34630, which is involved in UQ side chain biosynthesis in *Arabidopsis*, targets to both mitochondria and chloroplasts (Ducluzeau et al., 2012). Tomato SlDPS:GFP showed diffused fluorescence in *N. benthamiana* cells (Jones et al., 2013). The results are consistent with the report that solanesyl moiety was attached to benzoquinone through the Golgi/ER system in spinach leaves (Swiezewska et al., 1993). It indicates that the intermediates of UQ biosynthesis may move from one organelle to another in a cell (Ohara et al., 2004). Moreover, distinct function of *SmPPS1* and *SmPPS2* in *coq1* could be due to different subcellular localization (Jun et al., 2004; Ohara et al., 2010). For instance, adding a mitochondrial signal peptide to the N-terminus of *Arabidopsis* At1g78510 could result in the complementation of *S. pombe dlp1* and *dps1* mutant (Jun et al., 2004).

*SmPPS1* down-regulated *S. miltiorrhiza* plants with exhibited abnormal chloroplast development, abnormal trichome development, and varied leaf bleaching phenotypes. The phenotypes are similar to that found in *SlSPS*-silenced tomato transgenics (Jones et al., 2013). PQ is a cofactor of phytoene desaturation and is indirectly involved in the biosynthesis of carotenoids, which, together with PQ, are important antioxidants in protection of cell membranes against oxidative stress (Norris et al., 1995; Carol and Kuntz, 2001). Plants lacking PQ and carotenoids will be vulnerable to photo-oxidative damage and develop into photo-bleached seedlings (Chalker-Scott, 1999; Gould, 2004; Ksas et al., 2015; Liu and Lu, 2016). Abnormal chloroplast development in *SmPPS1* down-regulated plants could also be caused by photo-oxidative stress, since significant increasing of MDA content was observed. Homogentisate solanesyltransferase (HST) is the other key enzyme involved in PQ biosynthesis. Mutation of *HST* may also result in PQ deficiency and albino phenotype, such as *Arabidopsis pds2* mutant that has a 6bp-lesion in *AtHST* gene (Tian et al., 2007). However, *pds2* mutant showed stomata closure defects, which was not found in *SmPPS1* knockdown *S. miltiorrhiza* plants. It suggests the difference between *PPS* and *HST* down-regulated plants.

No visible phenotype changes were observed for *SmPPS2* transgenics, but down-regulation of *SmPPS2* might result in decrease of total UQ content, and up-regulation of *SmPPS2* might cause UQ accumulation. It suggests that *SmPPS2* is involved in UQ biosynthesis. The levels of *SmPPS2* transcripts in *SmPPS2* overexpression lines were significantly up-regulated from 22 to 52.5 times of WT. However, the content of total UQ was increased only by 52–63%. Similar phenomenon was observed for overexpression of At2g34630 in *Arabidopsis* (Ducluzeau et al., 2012). It indicates that overexpression of long-chain *PPS* gene has limited effects on plant UQ production. This is because long-chain PPS, which provides the prenyl side chain precursors for UQ structure, is not the rate-limiting enzyme of UQ biosynthesis (Liu and Lu, 2016). In contrast, overexpression of the rate-limiting enzyme gene *PPT/COQ2* could effectively improve UQ production in plants (Ohara et al., 2004; Stiff, 2010). From a biotechnological point of view, comprehensive approaches, such as simultaneously increasing side chain supply and activating rate-limiting steps, could be efficient ways to raise UQ content in transgenic plants (Kumar et al., 2012; Liu and Lu, 2016).

Taken together, this is the first report for UQ biosynthesis in a valuable medicinal model plant. The results provide useful information for further elucidation of PQ and UQ biosynthetic pathways and for the production of clinically significant UQ-10 through metabolic engineering and synthetic biology approaches.

## Data Availability

The datasets generated for this study can be found in NCBI GenBank, MH924998, JX090100.

## Author Contributions

SL conceived and designed the experiments. ML, YM, QD, XH, and MW performed the experiments. ML, YM, and SL wrote the manuscript. All authors approved the final version of the manuscript.

## Conflict of Interest Statement

The authors declare that the research was conducted in the absence of any commercial or financial relationships that could be construed as a potential conflict of interest.

## Footnotes

1 http://genes.mit.edu/GENSCAN.html

2 http://www.phylogeny.fr/

3 http://www.cbs.dtu.dk/services/TargetP/

## References

[B1] Altschul S. F. Gish W. Miller W. Myers E. W. Lipman D. J. (1990). Basic local alignment search tool. *J. Mol. Biol.* 215 403–410. 10.1006/jmbi.1990.9999 2231712

[B2] Altschul S. F. Madden T. L. Schäffer A. A. Zhang J. Zhang Z. Miller W. (1997). Gapped BLAST and PSI-BLAST: a new generation of protein database search programs. *Nucleic Acids Res.* 25 3389–3402. 10.1093/nar/25.17.3389 9254694 PMC146917

[B3] Asai K. Fujisaki S. Nishimura Y. Nishino T. Okada K. Nakagawa T. (1994). The identification of *Escherichia coli ispB* (*CEL*) gene encoding the octaprenyl diphosphate synthase. *Biochem. Biophys. Res. Commun.* 202 340–345. 10.1006/bbrc.1994.1933 8037730

[B4] Ashby M. N. Edwards P. A. (1990). Elucidation of the deficiency in two yeast coenzyme Q mutants. Characterization of the structural gene encoding hexaprenyl pyrophosphate synthetase. *J. Biol. Chem.* 265 13157–13164. 2198286

[B5] Bentinger M. Brismar K. Dallner G. (2007). The antioxidant role of coenzyme Q. *Mitochondrion* 7 S41–S50. 10.1016/j.mito.2007.02.006 17482888

[B6] Block A. Fristedt R. Rogers S. Kumar J. Barnes B. Barnes J. (2013). Functional modeling identifies paralogous solanesyl-diphosphate synthases that assemble the side chain of plastoquinone-9 in plastids. *J. Biol. Chem.* 288 27594–27606. 10.1074/jbc.m113.492769 23913686 PMC3779756

[B7] Bouvier F. Suire C. d’Harlingue A. Backhaus R. A. Camara B. (2000). Molecular cloning of geranyl diphosphate synthase and compartmentation of monoterpene synthesis in plant cells. *Plant J.* 24 241–252. 10.1046/j.1365-313x.2000.00875.x 11069698

[B8] Burge C. B. Karlin S. (1998). Finding the genes in genomic DNA. *Curr. Opin. Struct. Biol.* 8 346–354. 10.1016/S0959-440X(98)80069-9 9666331

[B9] Carol P. Kuntz M. (2001). A plastid terminal oxidase comes to light: implications for carotenoid biosynthesis and chlororespiration. *Trends Plant Sci.* 6 31–36. 10.1016/S1360-1385(00)01811-2 11164375

[B10] Chalker-Scott L. (1999). Environmental significance of anthocyanins in plant stress responses. *Photochem. Photobiol.* 70 1–9. 10.1111/j.1751-1097.1999.tb01944.x

[B11] Danesi F. Ferioli F. Caboni M. F. Boschetti E. Nunzio M. D. Verardo V. (2011). Phytosterol supplementation reduces metabolic activity and slows cell growth in cultured rat cardiomyocytes. *Br. J. Nutr.* 106 540–548. 10.1017/S0007114511000626 21554812

[B12] Deng Y. Lu S. (2017). Biosynthesis and regulation of phenylpropanoids in plants. *Crit. Rev. Plant Sci.* 36 257–290. 10.1080/07352689.2017.1402852

[B13] Dereeper A. Guignon V. Blanc G. Audic S. Buffet S. Chevenet F. (2008). Phylogeny.fr: robust phylogenetic analysis for the non-specialist. *Nucleic Acids Res.* 36 W465–W469. 10.1093/nar/gkn180 18424797 PMC2447785

[B14] Dhindsa R. S. Matowe W. (1981). Drought tolerance in two mosses: correlated with enzymatic defence against lipid peroxidation. *J. Exp. Bot.* 32 79–91. 10.1093/nar/gkn180 18424797 PMC2447785

[B15] Ducluzeau A. L. Wamboldt Y. Elowsky C. G. Mackenzie S. A. Schuurink R. C. Basset G. J. (2012). Gene network reconstruction identifies the authentic trans-prenyl diphosphate synthase that makes the solanesyl moiety of ubiquinone-9 in *Arabidopsis*. *Plant J.* 69 366–375. 10.1111/j.1365-313X.2011.04796.x 21950843

[B16] Fan W. J. Zhang M. Zhang H. X. Zhang P. (2012). Improved tolerance to various abiotic stresses in transgenic sweet potato (*Ipomoea batatas*) expressing spinach betaine aldehyde dehydrogenase. *PLoS One* 7:e37344. 10.1371/journal.pone.0037344 22615986 PMC3353933

[B17] Gao W. Hillwig M. L. Huang L. Cui G. Wang X. Kong J. (2009). A functional genomics approach to tanshinone biosynthesis provides stereochemical insights. *Org. Lett.* 11 5170–5173. 10.1021/ol902051v 19905026 PMC2776380

[B18] Gin P. Clarke C. F. (2005). Genetic evidence for a multi-subunit complex in coenzyme Q biosynthesis in yeast and the role of the *Coq1* hexaprenyl diphosphate synthase. *J. Biol. Chem.* 280 2676–2681. 10.1074/jbc.M411527200 15548532

[B19] Gould K. S. (2004). Nature’s swiss army knife: the diverse protective roles of anthocyanins in leaves. *J. Biomed. Biotechnol.* 5 314–320. 10.1155/S1110724304406147 15577195 PMC1082902

[B20] Hao G. Shi R. Tao R. Fang Q. Jiang H. Ji H. (2013). Cloning, molecular characterization and functional analysis of 1-hydroxy-2-methyl-2-(E)-butenyl-4-diphosphate reductase (HDR) gene for diterpenoid tanshinone biosynthesis in *Salvia miltiorrhiza* Bge. f. alba. *Plant Physiol. Biochem.* 70 21–32. 10.1016/j.plaphy.2013.05.010 23770591

[B21] Hayashi K. Ogiyama Y. Yokomi K. Nakagawa T. Kaino T. Kawamukai M. (2014). Functional conservation of coenzyme Q biosynthetic genes among yeasts, plants, and humans. *PLoS One* 9:e99038. 10.1371/journal.pone.0099038 24911838 PMC4049637

[B22] Hemmi H. Ikejiri S. Yamashita S. Nishino T. (2002). Novel medium-chain prenyl diphosphate synthase from the thermoacidophilic archaeon *Sulfolobus solfataricus*. *J. Bacteriol.* 184 615–620. 10.1128/JB.184.3.615-620.2002 11790729 PMC139513

[B23] Hirooka K. Bamba T. Fukusaki E. Kobayashi A. (2003). Cloning and kinetic characterization of *Arabidopsis thaliana* solanesyl diphosphate synthase. *Biochem. J.* 370 679–686. 10.1042/BJ20021311 12437513 PMC1223189

[B24] Hirooka K. Izumi Y. An C. I. Nakazawa Y. Fukusaki E. Kobayashi A. (2005). Functional analysis of two solanesyl diphosphate synthases from *Arabidopsis thaliana*. *Biosci. Biotechnol. Biochem.* 69 592–601. 10.1271/bbb.69.592 15784989

[B25] Hofgen R. Willmitzer L. (1988). Storage of competent cells for *Agrobacterium* transformation. *Nucleic Acids Res.* 16:9877. 10.1093/nar/16.20.9877 3186459 PMC338805

[B26] Hsieh F. L. Chang T. H. Ko T. P. Wang A. H. (2011). Structure and mechanism of an *Arabidopsis* medium/long-chain-length prenyl pyrophosphate synthase. *Plant Physiol.* 155 1079–1090. 10.1104/pp.110.168799 21220764 PMC3046570

[B27] Hu B. Jin J. Guo A.-Y. Zhang H. Luo J. Gao G. (2015). GSDS 2.0: an upgraded gene feature visualization server. *Bioinformatics* 31 1296–1297. 10.1093/bioinformatics/btu817 25504850 PMC4393523

[B28] Hunter C. T. Saunders J. W. Magallanes-Lundback M. Christensen S. A. Willett D. Stinard P. S. (2018). Maize *w3* disrupts homogentisata solanesyl transferase (*ZmHST*) and reveals a plastoquinone-9 independent path for phytoene desaturation and tocopherol accumulation in kernels. *Plant J.* 93 799–813. 10.1111/tpj.13821 29315977

[B29] Irish V. F. Sussex I. M. (1990). Function of *apetala-1* gene during *Arabidopsis* floral development. *Plant Cell* 2 741–753. 10.1105/tpc.2.8.741 1983792 PMC159927

[B30] Jones M. O. Perez-Fons L. Robertson F. P. Bramley P. M. Fraser P. D. (2013). Functional characterization of long-chain prenyl diphosphate synthases from tomato. *Biochem. J.* 449 729–740. 10.1042/BJ20120988 23126257

[B31] Jun L. Saiki R. Tatsumi K. Nakagawa T. Kawamukai M. (2004). Identification and subcellular localization of two solanesyl diphosphate synthases from *Arabidopsis thaliana*. *Plant Cell Physiol.* 45 1882–1888. 10.1093/pcp/pch211 15653808

[B32] Kai G. Liao P. Zhang T. Zhou W. Wang J. Xu H. (2010). Characterization, expression profiling and functional identification of a gene encoding geranylgeranyl diphosphate synthase from *Salvia miltiorrhiza*. *Biotechnol. Bioproc. Eng.* 15 236–245. 10.1007/s12257-009-0123-y

[B33] Kainou T. Okada K. Suzuki K. Nakagawa T. Matsuda H. Kawamukai M. (2001). Dimer formation of octaprenyl-diphosphate synthase (IspB) is essential for chain length determination of ubiquinone. *J. Biol. Chem.* 276 7876–7883. 10.1074/jbc.M007472200 11108713

[B34] Kamei M. Fujita T. Kanbe T. Sasaki K. Oshiba K. Otani S. (1986). The distribution and content of ubiquinone in foods. *Int. J. Vitam. Nutr. Res.* 56 57–63. 10.1016/0006-2952(86)90238-8 3710719

[B35] Kawamukai M. (2009). Biosynthesis and bioproduction of coenzyme Q10 by yeasts and other organisms. *Biotechnol. Appl. Biochem.* 53 217–226. 10.1042/BA20090035 19531029

[B36] Kim E. H. Lee D. W. Lee K. R. Jung S. J. Jeon J. S. Kim H. U. (2017). Conserved function of fibrillin5 in the plastoquinone-9 biosynthetic pathway in *Arabidopsis* and Rice. *Front. Plant Sci.* 8:1197. 10.3389/fpls.2017.01197 28751900 PMC5507956

[B37] Ksas B. Becuwe N. Chevalier A. Havaux M. (2015). Plant tolerance to excess light energy and photooxidative damage relies on plastoquinone biosynthesis. *Sci. Rep* 5:10919. 10.1038/srep10919 26039552 PMC4454199

[B38] Kumar S. Hahn F. M. Baidoo E. Kahlon T. S. Wood D. F. McMahan C. M. (2012). Remodeling the isoprenoid pathway in tobacco by expressing the cytoplasmic mevalonate pathway in chloroplasts. *Metab. Eng.* 14 19–28. 10.1016/j.ymben.2011.11.005 22123257 PMC5767336

[B39] Lau V. W. Journoud M. Jones P. J. (2005). Plant sterols are efficacious in lowering plasma LDL and non-HDL cholesterol in hypercholesterolemic type 2 diabetic and nondiabetic persons. *Am. J. Clin. Nutr.* 81 1351–1358. 10.1093/ajcn/81.6.1351 15941886

[B40] Lichtenthaler H. K. (1987). Chlorophyll florescence signature of leave the autumnal chlorophyll breakdown. *J. Plant Physiol.* 131 101–110. 10.1016/S0176-1617(87)80271-7

[B41] Liu M. Lu S. (2016). Plastoquinone and ubiquinone in plants: biosynthesis, physiological function and metabolic engineering. *Front. Plant Sci.* 7:1898. 10.3389/fpls.2016.01898 28018418 PMC5159609

[B42] Livak K. J. Schmittgen T. D. (2008). Analyzing real-time PCR data by the comparative CT method. *Nat. Protoc.* 3 1101–1108. 10.1038/nprot.2008.73 18546601

[B43] Long M. Rosenberg C. Gilbert W. (1995). Intron phase correlations and the evolution of the intron/exon structure of genes. *Proc. Natl. Acad. Sci. U.S.A.* 92 12495–12499. 10.1073/pnas.92.26.12495 8618928 PMC40384

[B44] Ma Y. Yuan L. Wu B. Li X. Chen S. Lu S. (2012). Genome-wide identification and characterization of novel genes involved in terpenoid biosynthesis in *Salvia miltiorrhiza*. *J. Exp. Bot.* 63 2809–2823. 10.1093/jxb/err466 22291132 PMC3346237

[B45] Marchler-Bauer A. Lu S. Anderson J. B. Chitsaz F. Derbyshire M. K. DeWeese-Scott C. (2011). CDD: a conserved domain database for the functional annotation of proteins. *Nucleic Acids Res.* 39 D225–D229. 10.1093/nar/gkq1189 21109532 PMC3013737

[B46] Mattila P. Kumpulainen J. (2001). Coenzymes Q9 and Q10: contents in foods and dietary intake. *J. Food Compos. Anal.* 14 409–417. 10.1006/jfca.2000.0983

[B47] Mei X. D. Cao Y. F. Che Y. Y. Li J. Shang Z. P. Zhao W. J. (2019). Danshen: a phytochemical and pharmacological overview. *Chin. J. Nat. Med.* 17 59–80. 10.1016/S1875-5364(19)30010-X 30704625

[B48] Nicholas K. B. Nicholas H. B. Jr. Deerfield D. W. I. (1997). GeneDoc: analysis and visualization of genetic variation. *Embnet News* 4 14–17.

[B49] Norris S. R. Barrette T. R. DellaPenna D. (1995). Genetic dissection of carotenoid synthesis in Arabidopsis defines plastoquinone as an essential component of phytoene desaturation. *Plant Cell* 7 2139–2149. 10.2307/3870157 8718624 PMC161068

[B50] Ohara K. Kokado Y. Yamamoto H. Sato F. Yazaki K. (2004). Engineering of ubiquinone biosynthesis using the yeast *coq2* gene confers oxidative stress tolerance in transgenic tobacco. *Plant J.* 40 734–743. 10.1111/j.1365-313x.2004.02246.x 15546356

[B51] Ohara K. Sasaki K. Yazaki K. (2010). Two solanesyl diphosphate synthases with different subcellular localizations and their respective physiological roles in *Oryza sativa*. *J. Exp. Bot.* 61 2683–2692. 10.1093/jxb/erq103 20421194 PMC2882263

[B52] Ohara K. Yamamoto K. Hamamoto M. Sasaki K. Yazaki K. (2006). Functional characterization of *OsPPT1*, which encodes *p*-hydroxybenzoate polyprenyltransferase involved in ubiquinone biosynthesis in *Oryza sativa*. *Plant Cell Physiol.* 47 581–590. 10.1093/pcp/pcj025 16501255

[B53] Phatthiya A. Takahashi S. Chareonthiphakorn N. Koyama T. Wititsuwannakul D. Wititsuwannakul R. (2007). Cloning and expression of the gene encoding solanesyl diphosphate synthase from *Hevea brasiliensis*. *Plant Sci.* 172 824–831. 10.1016/j.plantsci.2006.12.015

[B54] Pyo Y. H. (2010). Coenzyme Q10 and Q9 content in 6 commercial vegetable oils and their average daily intakes in Korea. *Food Sci. Biotechnol.* 19 837–841. 10.1007/s10068-010-0118-7

[B55] Rodríguez-Acuña R. Brenne E. Lacoste F. (2008). Determimation of coenzyme Q10 and Q9 in vegetable oils. *J. Agric. Food Chem.* 56 6241–6245. 10.1021/jf800103e 18616270

[B56] Saiki R. Nagata A. Uchida N. Kainou T. Matsuda H. Kawamukai M. (2003). Fission yeast decaprenyl diphosphate synthase consists of Dps1 and the newly characterized Dlp1 protein in a novel heterotetrameric structure. *Eur. J. Biochem.* 270 4113–4121. 10.1046/j.1432-1033.2003.03804.x 14519123

[B57] Schwab R. Ossowski S. Riester M. Warthmann N. Weigel D. (2006). Highly specific gene silencing by artificial microRNAs in *Arabidopsis*. *Plant Cell* 18 1121–1133. 10.1105/TPC.105.039834 16531494 PMC1456875

[B58] Shi R. Yang C. Lu S. Sederoff R. Chiang V. L. (2010). Specific down-regulation of PAL genes by artificial microRNAs in *Populus trichocarpa*. *Planta* 232 1281–1288. 10.1007/s00425-010-1253-3 20725738

[B59] Shukla S. Dubey K. K. (2018). CoQ10 a super-vitamin: review on application and biosynthesis. *3 Biotech* 8:249. 10.1007/s13205-018-1271-6 29755918 PMC5943198

[B60] Song J. Y. Luo H. M. Li C. F. Sun C. Xu J. Chen S. L. (2013). *Salvia miltiorrhiza* as medicinal model plant. *Yao Xue Xue Bao* 48 1099–1106. 24133975

[B61] Sparkes I. A. Runions J. Kearns A. Hawes C. (2006). Rapid, transient expression of fluorescent fusion proteins in tobacco plants and generation of stably transformed plants. *Nat. Protoc.* 1 2019–2025. 10.1038/nprot.2006.286 17487191

[B62] Stiff M. R. (2010). *Coenzyme Q10 Biosynthesis in Plants: is the Polyprenyltransferase an Appropriate Gene Target for the Increased Production of CoQ?.* Ph.D thesis, North Carolina State University, Raleigh.

[B63] Suzuki K. Okada K. Kamiya Y. Zhu X. F. Nakagawa T. Kawamukai M. (1997). Analysis of the decaprenyl diphosphate synthase (*dps*) gene in fission yeast suggests a role of ubiquinone as an antioxidant. *J. Biochem.* 121 496–505. 10.1093/oxfordjournals.jbchem.a021614 9133618

[B64] Swiezewska E. Dallner G. Andersson B. Ernster L. (1993). Biosynthesis of ubiquinone and plastoquinone in the endoplasmic reticulum-Golgi membranes of spinach leaves. *J. Biol. Chem.* 268 1494–1499. 8419349

[B65] Thompson J. D. Higgins D. G. Gibson T. J. (1994). CLUSTAL W: improving the sensitivity of progressive multiple sequence alignment through sequence weighting, position-specific gap penalties and weight matrix choice. *Nucleic Acids Res.* 22 4673–4680. 10.1007/978-1-4020-6754-9_3188 7984417 PMC308517

[B66] Tian L. DellaPenna D. Dixon R. A. (2007). The *pds2* mutation is a lesion in the *Arabidopsis* homogentisate solaneyltransferase gene involved in plastoquinone biosynthesis. *Planta* 226 1067–1073. 10.1007/s00425-007-0564-5 17569077

[B67] van Schie C. C. Ament K. Schmidt A. Lange T. Haring M. A. Schuurink R. C. (2007). Geranyl diphosphate synthase is required for biosynthesis of gibberellins. *Plant J.* 52 752–762. 10.1111/j.1365-313X.2007.03273.x 17877699

[B68] Wang K. Ohnuma S. (1999). Chain-length determination mechanism of isoprenyl diphosphate synthases and implications for molecular evolution. *Trends Biochem. Sci.* 24 445–451. 10.1016/S0968-0004(99)01464-4 10542413

[B69] Wang M. Deng Y. Shao F. Liu M. Pang Y. Li C. (2017). *ARGONAUTE* genes in *Salvia miltiorrhiza*: identification, characterization, and genetic transformation. *Methods Mol. Biol.* 1640 173–189. 10.1007/978-1-4939-7165-7_12 28608342

[B70] Wang L. Ma R. Liu C. Liu H. Zhu R. Guo S. (2017). *Salvia miltiorrhiza*: a potential red light to the development of cardiovascular diseases. *Curr. Pharm. Des.* 23 1077–1097. 10.2174/1381612822666161010105242 27748194 PMC5421141

[B71] Xing B. Yang D. Liu L. Han R. Sun Y. Liang Z. (2018). Phenolic acid production is more effectively enhanced than tanshinone production by methyl jasmonate in *Salvia miltiorrhiza* hairy roots. *Plant Cell Tissue Org.* 134 119–129. 10.1007/s11240-018-1405-x

[B72] Xing S. Miao J. Li S. Qin G. Tang S. Li H. (2010). Disruption of the 1-deoxy-D-xylulose-5-phosphate reductoisomerase (*DXR*) gene results in albino, dwarf and defects in trichome initiation and stomata closure in *Arabidopsis*. *Cell Res.* 20 688–700. 10.1038/cr.2010.54 20404857

[B73] Xu H. Song J. Luo H. Zhang Y. Li Q. Zhu Y. (2016). Analysis of the genome sequence of the medicinal plant *Salvia miltiorrhiza*. *Mol. Plant* 9 949–952. 10.1016/j.molp.2016.03.010 27018390 PMC5517341

[B74] Zbierzak A. M. Kanwischer M. Wille C. Vidi P. A. Giavalisco P. Lohmann A. (2010). Intersection of the tocopherol and plastoquinol metabolic pathways at the plastoglobule. *Biochem. J.* 425 389–399. 10.1042/BJ20090704 19843012

[B75] Zhang G. Tian Y. Zhang J. Shu L. Yang S. Wang W. (2015). Hybrid de novo genome assembly of the Chinese herbal plant danshen (*Salvia miltiorrhiza* Bunge). *Gigascience* 4:62. 10.1186/s13742-015-0104-3 26673920 PMC4678694

[B76] Zhang L. Lu S. (2017). Overview of medicinally important diterpenoids derived from plastids. *Mini Rev. Med. Chem.* 17 988–1001. 10.2174/1389557516666160614005244 27297674

