# Supplementary material for: Functional Analysis of Polyprenyl Diphosphate Synthase Genes Involved in Plastoquinone and Ubiquinone Biosynthesis in Salvia miltiorrhiza
Source: Front Plant Sci. 2019 Jul 9;10:893. doi: 10.3389/fpls.2019.00893 (PMC6629958; doi:10.3389/fpls.2019.00893)
Supplement: Supplementary file 1 [file Table_1.DOCX]

Supplementary Material

**Functional analysis of polyprenyl diphosphate synthase genes involved in plastoquinone and ubiquinone biosynthesis in *Salvia miltiorrhiza***

Miaomiao Liu^1,†^, Yimian Ma^1,†^, Qing Du^1,2^, Xuemin Hou^1^, Meizhen Wang^1^, Shanfa Lu^1,*^

1. Institute of Medicinal Plant Development, Chinese Academy of Medical Sciences & Peking Union Medical College, No.151, Malianwa North Road, Haidian District, Beijing 100193, China

2. Key Laboratory for Tibet Plateau Phytochemistry of Qinghai Province, College of Pharmacy, Qinghai Nationalities University, Xining, Qinghai 810007, China

**TABLE S1** Primers used for 5′ and 3′ RACE of *SmPPS1* and *SmPPS2*.

| **Primer name** | **Sequences (5′ to 3′)** |
| --- | --- |
| SmPPS1-R3 | 5′-GCTTCTGGAGTCTTCGTGGAGAAGA-3′ |
| SmPPS1-F1 | 5′-GGTTCTCTTGAGGAGGCCATTGAGA-3′ |
| SmPPS1-R2 | 5′-CTCCGCCACGCCTATTCACATTCT-3′ |
| SmPPS1-F3 | 5′-GAGTCCCTTTAGGCTATCGCTGGAA-3′ |
| SmPPS2-R3 | 5′-GTTGCCCAACCGTACTAATGGCATT-3′ |
| SmPPS2-F2 | 5′-GCATGCTAGGCTTGCATCAGCTGCAA-3′ |
| SmPPS2-R2 | 5′-GGATTGCGAAAGTGGGAGGAATGCT-3′ |
| SmPPS2-F3 | 5′-GACGAGGTTGTGCAGAGGTCGAT-3′ |

**TABLE S2** Primers used for cloning of full-length *SmPPS1* and *SmPPS2* cDNAs.

| **Primer name** | **Sequences (5′ to 3′)** |
| --- | --- |
| PPS1-F | 5′-CCATGATGTCGCTGACATGTAGTAATCT-3′ |
| PPS1-R | 5′-GGTCATTTTATCCGTTCCAAATTATAGT-3′ |
| PPS2-F | 5′-GGGTCTACTCGTCTTTGGGGTGTA-3′ |
| PPS2-R | 5′-CGATTGCAGCTGATGCAAGCCTAGCA-3′ |

**TABLE S3** Primers used for qRT-PCR.

| **Primer name** | **Sequences (5′ to 3′)** |
| --- | --- |
| UBQ-Fq | 5′-AGATGGGCGGACACTTGCTGATTA-3′ |
| UBQ-Fq | 5′-ACTCTCCACCTCCAAAGTGATGGT-3′ |
| PPS1-Fq | 5′-ACCAAGGGATCGGCCATTTTCAGT-3′ |
| PPS1-Rq | 5′-TCCCAGCTGCTCTGCTGACTGAGT-3′ |
| PPS2-Fq | 5′-ATACAAGGCGGGGTATTGGCTCTTT-3′ |
| PPS2-Rq | 5′- TGCATGGTCTCACCAGTGACCAGA-3′ |
